# Variability of crossing phase in older people with Parkinson’s disease is dependent of obstacle height

**DOI:** 10.1038/s41598-018-33312-2

**Published:** 2018-10-05

**Authors:** Lucas Simieli, Fabio Augusto Barbieri, Diego Orcioli-Silva, Ellen Lirani-Silva, Victor Spiandor Beretta, Paulo Cezar Rocha dos Santos, Lilian Teresa Bucken Gobbi

**Affiliations:** 1 São Paulo State University (Unesp) - Campus Rio Claro, Posture and Gait Studies Laboratory (LEPLO), Department of Physical Education, Rio Claro, Brazil; 2 São Paulo State University (Unesp) - Campus Bauru, Human Movement Research Laboratory (MOVI-LAB), Department of Physical Education, Bauru, Brazil

## Abstract

Our aim was investigating variability in spatiotemporal parameters and kinetics of obstacle avoidance steps of different height obstacles in people with Parkinson’s disease (PD) and healthy older people. Twenty-eight participants have included (15 PD – stage disease: 2.1 ± 0.4 pts) and 13 healthy older people (control group). Each subject performed 10 trials of the following tasks: low obstacle, intermediate obstacle and high obstacle. The presentation order was randomized by block for each condition and participant. The spatiotemporal parameters was collected by GAITRite. An optoelectronic system (Optotrak Northern Digital Inc.) with 100 Hz of frequency was used to collect obstacle parameters. The kinect parameters (propulsion impulse and braking impulse) were acquire through two force plates (AccuGait), with a frequency of acquisition 200 Hz. Intersteps variability was calculated throughout mean values, standard deviation and coefficient of variation of two obstacle avoidance steps for each trial. PD group presented greater variability than control group on vertical and horizontal distances to the obstacle. Obstacle height did not change kinect’s parameters for both groups. The combination of task complexity (obstacle height) and disease impairments (gait alteration, loss of balance, etc) contributing for greater variability of Parkinson’s group. Besides, low obstacle and high obstacle seem to exacerbate variability of distance between obstacle and foot.

## Introduction

Dealing with obstacle and complex environments are a challenging daily life tasks and represents the most cause of falls in older^1^. We have found in our previous study that both people with PD and neurological healthy individuals increased their step-to-step and walking variability in the steps preceding low obstacle avoidance compared to other obstacle heights (intermediate and high obstacles), mainly in last step (n-1) before obstacle avoidance^2^. However, this study was not focused on how obstacle height could affect obstacle avoidance itself. Vitório *et al*.^3^ observed that during approach phase, PD presented a shorter step length (considered a bad behavior) but, during high obstacle crossing, they could increase toe-clearance. This behavior could indicate a different behavior and brain control for these two parts of obstacle avoidance (approaching phase and obstacle crossing). Moreover, obstacle crossing is a crucial point that happens daily with different height and shapes. Foot positioning in the last step before obstacle avoidance and after obstacle avoidance (distance between foot and obstacle and toe clearance), the impulse to cross the obstacle and toe clearance could be an important information to avoid trips during obstacle avoidance in people with PD^1^. Low variability in these parameters during obstacle avoidance could indicate accuracy and security of the system that controls gait^4,5^. The present study is the first study that investigated the variability of different parameters of the avoidance step in people with PD, especially considering obstacle height manipulations.

The obstacle height seems an important aspect in the strategy (adjustments) to avoid an obstacle. When the obstacle height is manipulated there is an increased in the motor requirement and planning, which demands more of the corporal and navigational system^6,7^. However, during approaching phase, our previous study^2^ showed, as expected, different behavior according obstacle height. During approaching phase for low obstacles, people with PD and their healthy-matched control increase the variability for the last foot positioning before obstacle avoidance. In other words, for the lowest one, they changed their gait very closer to the obstacle^2^. Thus, due to different heights of obstacle that are facing daily, obstacle crossing could be considered a very dangerous situation that could leading to fall. For example, higher obstacle requires conservative strategy in people with PD, which increase the toe clearance of both limb (leading and trailing limbs) and the step duration of the crossing step^3,8^. Probably the deficits in the spatial-temporal^3,9^ and neuromuscular^10^ adjustments, executive function^11^, attention^11,12^ and perceptual system^10^ presented by people with PD require more time-to-time adjustments^13^ to avoid an obstacle. Impairments on basal ganglia connections like pedunculopontine nucleus, that is related with control and voluntary movements^11^, may explain the deficits on postural control instability, walking deficits (slower steps, shorter steps, greater step duration), deficits on attention and learning problems, correction of movements (variability))^14,15^ in older people with PD. In addition, people with PD have difficult to synchronize visual and kinesthetic stimulus^16,17^, impairing the ability to adjust themselves when the context is manipulated. Therefore, the influence of obstacle height on variability of crossing step parameters could help to understand the pathology of gait in people with PD.

The aim of this study, therefore, was to investigate the effects of obstacle height (low, intermediate and high obstacle) on variability of spatial–temporal of trailing and leading limb and impulses of crossing step in people with PD and neurological healthy individuals. According our previous study^2^, we expected that the variability of gait parameters will be greater in the low obstacle avoidance in people with PD since this condition present a greater adjustment range than higher obstacle.

## Methods

### Participants

Data for this study were obtained in the same experiment as those published previously in Simieli *et al*.^2^. Older people with PD were selected from the Physical Activity Program for Patients with PD (PROPARKI Group - UNESP – Rio Claro – Brazil) database (more than one hundred individuals). Older neurologically healthy individuals were selected from the database of the Physical Activity Program for Older People (PROFIT - UNESP - Rio Claro). Therefore, the following exclusion criteria were analyzed previously for both groups: age under 60, cognitive decline, history of orthopedic problems (if the participant use any walking aid, we excluded from the sample), vision (glaucoma, cataract) and vestibular (dizziness, labyrinthitis) that prevented performance of the experimental protocol. Participants with diabetes mellitus were also excluded, since plantar sensitivity may be altered and thereby, impair gait^2^. Any visual problems that could be corrected with glasses, were included on the sample. In addition, for older people with PD, the individual was required to be under dopaminergic medication treatment and in a stage of PD up to III according to the Hoehn & Yahr disability Scale^18,19^. Furthermore, an experienced neurologist evaluated and diagnosed the older people with PD according to the London Brain Bank – a guideline for diagnosis^20^.

### Clinical and walking with obstacle crossing evaluations

Both clinical and gait evaluations of the PD group were performed in an “ON” state of medication, about an hour after taking the dopaminergic medication. The following clinical scale were applied in older people with PD: Unified Parkinson’s Disease Rating Scale – UPDRS^19^; the Hoehn and Yahr score^18^; and Mini Mental State Examination^21^ (performed by both groups). The analysis with G*Power software showed that a sample size of at least 14 individuals (7 in each group) was needed for an 95% probability to detect a difference of 20% between the groups for the primary outcome with a type I error of 0.05, based on previously published data (Hausdorff and colleagues^22^).

**Figure 1:**
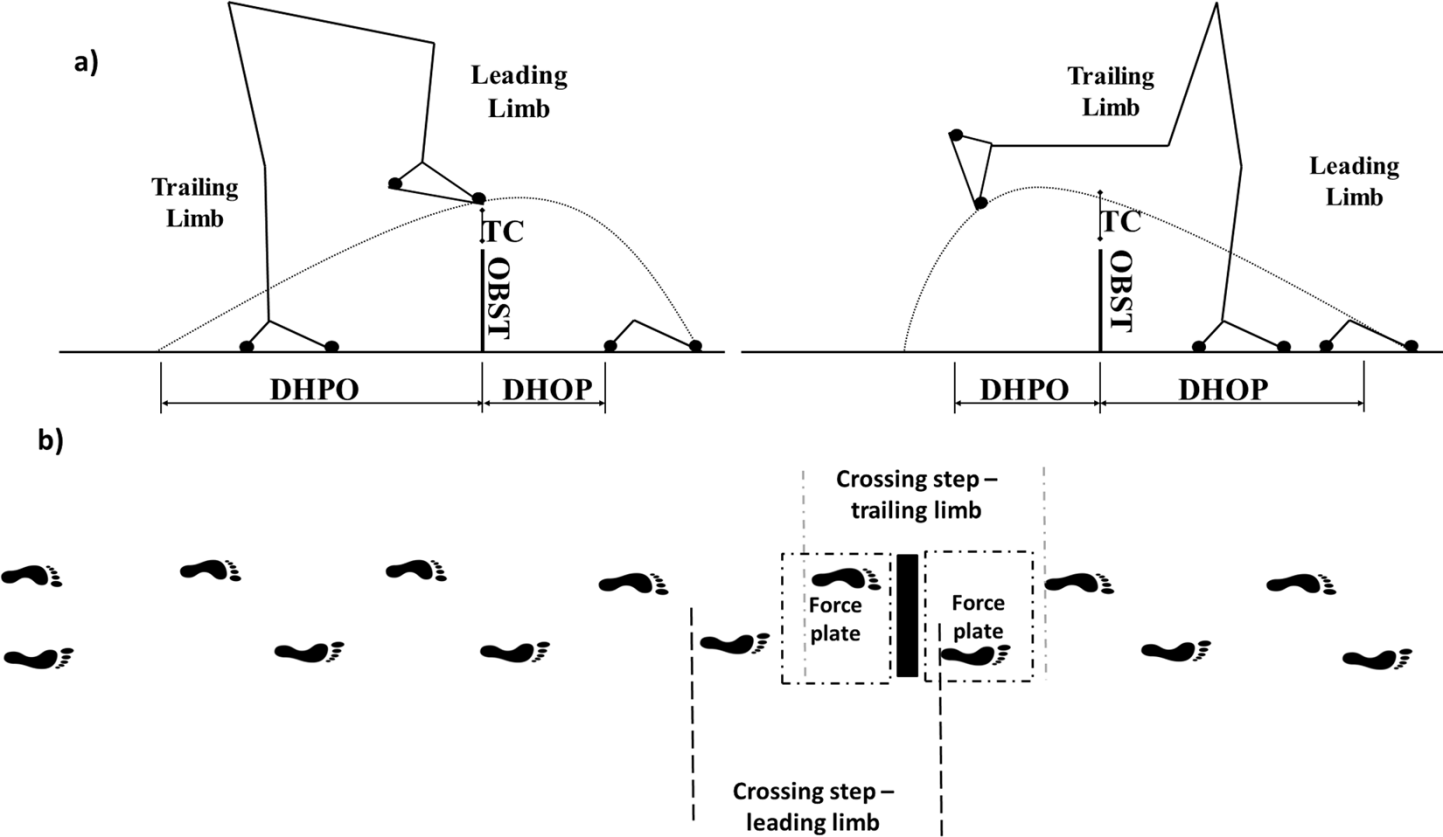
Schematic of steps analyzed during the task. (**a**) Illustrate the obstacle crossing parameters^3^. (**b**) Illustrates all steps performed and steps analyzed.

Participants were instructed to walk at their preferred speed until the end of the walkway (8 m). Ten trials of following walking conditions were performed by participants: low obstacle avoidance, intermediate obstacle avoidance and high obstacle avoidance. Thus, each participant performed 30 trials in total. The trials were performed in blocks in a randomized order for all participants. The obstacle was positioned in the center of the walkway (4 m from the start position), which height was customized for each individual. High obstacle was height equal to half the knee height (if the half knee was greater than 48.5 cm, the obstacle height was 25 cm, if lower than 48.5 cm, the obstacle height was 20 cm). Low obstacle was height equal to the ankle height (if the ankle height was greater than 7 cm, the obstacle height was 10 cm; if lower than 7 cm, the obstacle height was 5 cm). Intermediate obstacle was height equal to half the sum of the high obstacle height and low obstacle height. The obstacle width and length were 60 cm and 3 cm, respectively, independent of obstacle height. The start point was adjusted to ensure comfortable crossing with the right leg. In addition, the participants were instructed to avoid contact with the obstacle in trials where the obstacle was present (see Fig. 1).

The spatial-temporal parameters were acquired through GAITRite® (CIR System, Clifton, NJ, USA) and three-dimensional optoelectronic system (OPTOTRAK Certus), positioned orthogonal to the plane of progression to the right of the walkway, both with frequency of 100 samples/s. Four infrared emitters were placed over the following anatomical points: lateral face of calcaneus and head of the fifth metatarsus of the right limb, and medial face of calcaneus and head of the first metatarsus of the left limb. The data were filtered with a fifth order low-pass Butterworth filter with a cut-off frequency of 6 Hz). Two force plates (200 samples/s) – 50 × 50 cm (AccuGait, Advanced Mechanical Technologies, Boston, MA) were used to collect ground force reaction. The first force plate was placed before obstacle avoidance (force plate was stepped with trailing limb – left limb) and the second force plate was placed after obstacle (force plate was stepped with leading limb – right limb). Force plate data were filtered with a 4th order filter with a cutoff frequency of 16 Hz and the magnitude of the ground reaction force was normalized by body weight.

### Data analysis

The parameters were calculated in Matlab (Version 7.0 – Math Works, Inc.). The following parameters were analyzed: step length, step duration, step velocity, step width, double support time (expressed by percentage of step duration), single support time (expressed by percentage of step duration), toe clearance of the leading and trailing limbs (vertical distance from foot to obstacle), horizontal distance from the leading and trailing limb to the obstacle before and after obstacle crossing, and braking and propulsive vertical impulses. These parameters were calculated during obstacle avoidance steps from leading limb (right limb) and trailing limb (left limb).

The variability of these parameters was calculated by the average values and standard deviations for each step for each participant, followed by the coefficient of variation of each step in each condition^2,4,23,24^. We calculated the variability of step of leading limb and trailing limb (2 steps).

### Statistical analysis

The data of interest was statistically analyzed using SPSS 15.0 software for Windows with a significance level maintained at 0.05. The Shapiro-Wilk and Levene’s tests were used to verify the normal distribution of data and homogeneity of variance, respectively. The variability of parameters was compared through two-way ANOVAs for group (PD group x control group) and condition (low obstacle x intermediate obstacle x high obstacle), with repeated measures for condition. We also compared the trials to verify if they have a learning effect. Bonferroni post-hoc tests were performed to identify differences when the ANOVA revealed significant interactions.

## Results

Twenty-eight participated in this study, who were distributed in two groups: 15 older people with PD (70.66 ± 6.55 years old; 1.63 ± 0.07 m; 70.61 ± 9.82 kg; Hoehn & Yahr = 2.1 ± 0.4 pts; UPDRS-motor = 24.75 ± 11.25 pts; 8 men) – PD group; and 13 older neurologically healthy individuals (71.53 ± 5.42 years old; 1.59 ± 0.08 m; 70.50 ± 15.49 kg; 7 men) – control group. Individuals of both group have no cognitive deficits, which was analyzed by the Mini Mental State Examination (PD group – 28.26 ± 1.66 pts; control group – 28.46 ± 1.63 pts). There are no different between groups for age (p = 0.76), body weight (p = 0.98), height (p = 0.20) and Mini Mental State Examination (p = 0.76). We also performed a chi-square for sex frequencies in each group, and there were no differences (PD group Χ^2^ = 0.067, p = 0.796; Control group Χ^2^ = 0.077, p = 0.782). Moreover, we have did an analysis to verify if they had any learning effect among trials. For this, we compared the values of first trial, second, third until the last one. We did not find any difference between the trials and, consequently, no learning effects for each group (we compared (ANOVA factor for group and trial sequence) first trial to second trial, third, fourth, until tenth (p > 0.05.). There were no freezing episodes during the experiment.

The means values of parameters for each group and according each condition are presented in Table 1.

**Table 1:** Mean (standard deviation) of obstacle avoidance. PC: body weight (standard deviation) of obstacle avoidance; DHPO: horizontal distance between foot and obstacle before the obstacle; DHOP: horizontal distance between foot and obstacle after the obstacle; TC: toe-clearance.

Spatiotemporal parameters												
	Low obstacle				Intermediate obstacle				High obstacle			
	Right leg (leading limb)		Left leg (trailing limb)		Right leg (leading limb)		Left leg (trailing limb)		Right leg (leading limb)		Left leg (trailing limb)	
	Control	PD	Control	PD	Control	PD	Control	PD	Control	PD	Control	PD
Step length (cm)	67.69 (7.69)	63.68 (7.02)	59.20 (11.63)	52.30 (9.82)	67.30 (8.53)	62.81 (7.06)	59.53 (10.23)	54.67 (7.24)	66.12 (7.69)	63.43 (7.31)	59.68 (13.05)	52.00 (8.11)
Duration (s)	0.63 (0.07)	0.70 (0.09)	0.62 (0.08)	0.67 (0.07)	0.66 (0.08)	0.74 (0.09)	0.66 (0.09)	0.73 (0.09)	0.70 (0.09)	0.78 (0.08)	0.71 (0.10)	0.77 (0.09)
Width (cm)	11.38 (5.01)	11.61 (3.02)	13.97 (3.63)	13.20 (2.99)	10.70 (5.01)	12.10 (3.93)	14.88 (3.83)	13.03 (2.77)	11.78 (6.03)	12.26 (3.16)	14.44 (5.27)	14.21 (2.78)
Double support (%)	15.78 (1.67)	17.40 (1.37)	18.02 (2.12)	21.41 (2.64)	14.80 (1.24)	15.82 (1.64)	18.00 (2.12)	20.83 (2.22)	13.93 (1.58)	14.77 (1.67)	16.55 (2.96)	20.75 (2.10)
Single support (%)	84.21 (1.67)	82.59 (1.37)	75.92 (22.66)	78.58 (2.64)	85.19 (1.24)	84.17 (1.64)	81.99 (2.12)	80.47 (2.57)	86.06 (1.58)	85.22 (1.67)	83.44 (2.96)	81.19 (3.28)
Step velocity (cm/s)	109.01 (18.10)	92.20 (16.94)	96.13 (22.92)	78.91 (18.45)	103.29 (18.37)	86.01 (15.99)	91.59 (21.34)	75.99 (13.80)	95.61 (15.43)	82.30 (14.16)	85.36 (22.18)	68.69 (14.14)
**Kinects Parameters**												
	**Before obstacle**						**After obstacle**					
	**Low obstacle**		**Intermediate obstacle**		**High obstacle**		**Low obstacle**		**Intermediate obstacle**		**High obstacle**	
	**Control**	**PD**	**Control**	**PD**	**Control**	**PD**	**Control**	**PD**	**Control**	**PD**	**Control**	**PD**
Braking (%pc/s)	0.18 (0.08)	0.12 (0.04)	0.19 (0.02)	0.13 (0.04)	0.21 (0.03)	0.14 (0.04)	0.20 (0.05)	0.10 (0.06)	0.21 (0.06)	0.22 (0.08)	0.22 (0.08)	0.23 (0.09)
Propulsion (%pc/s)	0.11 (0.06)	0.09 (0.03)	0.12 (0.07)	0.09 (0.04)	0.13 (0.07)	0.10 (0.04)	0.10 (0.06)	0.20 (0.08)	0.11 (0.08)	0.11 (0.09)	0.12 (0.09)	0.11 (0.06)
**Horizontal distances and toe-clearance**												
	**Control Group**						**PD group**					
	**Leading limb**			**Trailing limb**			**Leading limb**			**Trailing limb**		
	**DHPO**	**DHOP**	**TC**	**DHPO**	**DHOP**	**TC**	**DHPO**	**DHOP**	**TC**	**DHPO**	**DHOP**	**TC**
Low obstacle	73.46 (14.59)	27.31 (6.16)	23.24 (5.07)	12.89 (4.31)	60.22 (10.23)	3.85 (2.24)	58.44 (9.40)	23.69 (4.04)	23.61 (7.94)	13.60 (4.79)	65.12 (9.68)	5.64 (4.20)
Intermediate obstacle	73.74 (12.54)	28.70 (6.90)	19.51 (5.42)	12.42 (4.45)	58.88 (10.13)	3.63 (1.42)	58.29 (10.27)	22.98 (3.88)	20.23 (19.51)	13.66 (3.86)	64.78 (7.47)	3.50 (2.66)
High obstacle	69.38 (10.07)	27.11 (5.56)	13.06 (5.90)	24.63 (4.35)	72.65 (9.74)	7.84 (3.89)	58.47 (8.07)	23.45 (4.01)	14.72 (8.77)	13.77 (4.04)	66.55 (5.30)	7.06 (2.66)

### Variability of gait parameters

**Figure 2:**
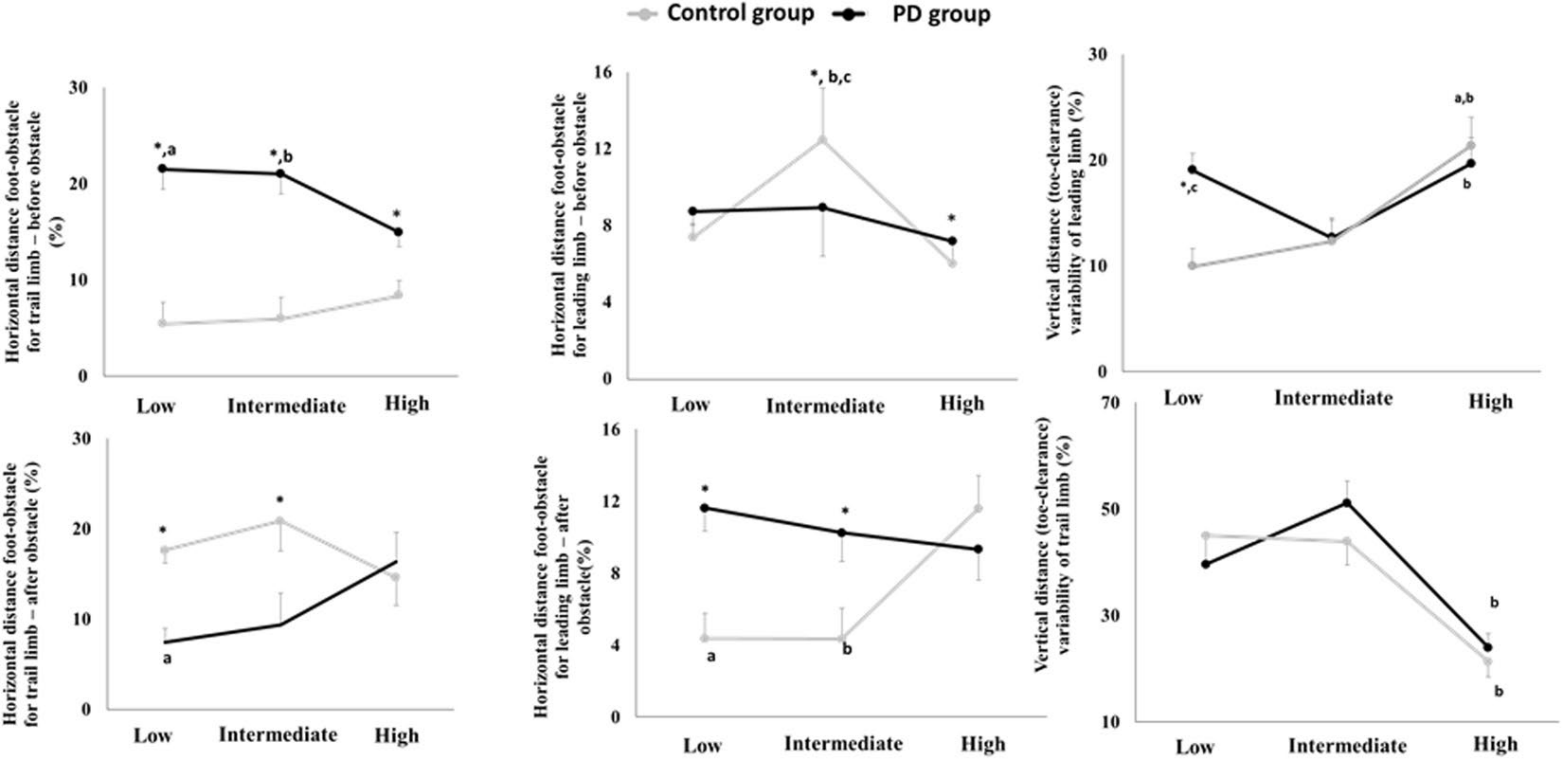
Variability of kinects parameters for each group in each condition.

ANOVA did not indicate main effects of group and group*condition interaction for kinetic parameters of force plate placed after the obstacle (F_4,23_ = 0.342, p = 0.323). The ANOVA indicated a condition effect for force plate placed before obstacle for propulsion impulse (F_4,23_ = 4,720, p = 0.006). In addition, high obstacle presented greater variability of propulsive impulse compared to intermediate obstacle (p = 0.003 – Fig. 2).

The ANOVA indicated group*condition interaction. PD group presented greater variability of horizontal distance from the trailing limb to the obstacle before obstacle crossing (F_2,52_ = 1.169, p < 0.001) compared to control group in all obstacle height. In addition, PD group increased variability of horizontal distance from the leading limb to the obstacle after obstacle crossing and decreased variability of horizontal distance from the trailing limb to the obstacle after obstacle crossing compared to control group during low (F_2,52_ = 7.753, p < 0.001) and intermediate (F_2,52_ = 7.753, p < 0.01) obstacle avoidance. Furthermore, during low obstacle avoidance, PD group revealed greater variability of leading limb toe-clearance compared to control group (F_2,52_ = 6.345, p < 0.001). Finally, PD group increased variability of leading limb toe-clearance during low (F_2,52_ = 6.345, p < 0.002) and high (F_2,52_ = 6,345 p < 0.004) obstacle avoidance compared to intermediate obstacle avoidance while control group increased variability of leading limb toe-clearance during high obstacle avoidance compared to low and intermediate obstacle avoidance (F_2,52_ = 6.345, p < 0.001).

**Figure 3:**
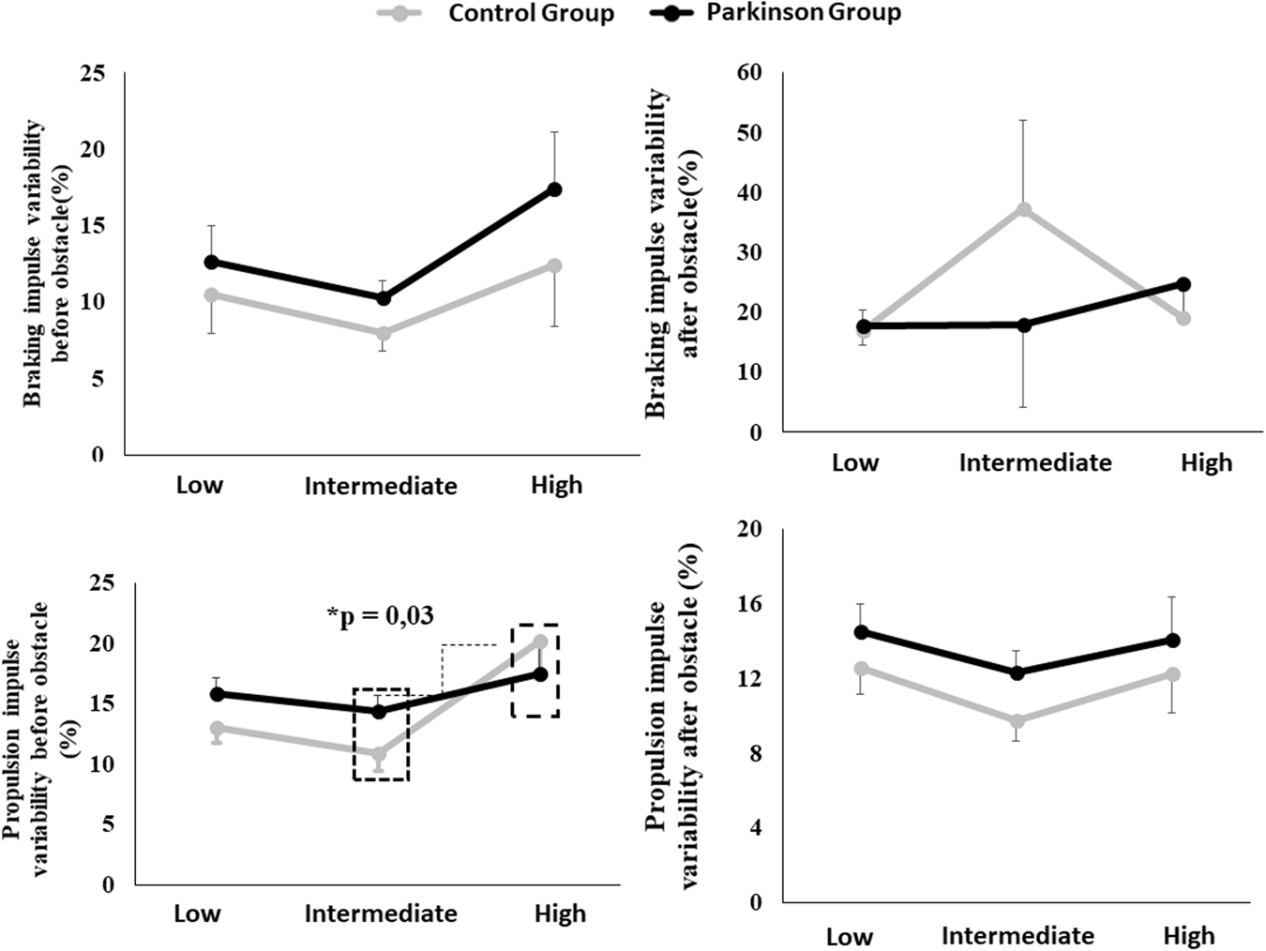
Group*obstacle variability for horizontal and vertical foot-obstacle distance before and after obstacle avoidance for leading and trailing limb. (**a**) Difference between low and high obstacle avoidance; (**b**) Difference between intermediate and high obstacle avoidance; (**c**) difference between low and intermediate obstacle. *Difference between people with PD and control group.

For group (Fig. 3), older people with PD presented greater variability of horizontal distance from the trailing limb to the obstacle before obstacle crossing (F_4,23_ = 10.201, p < 0.001) and horizontal distance from the leading limb to the obstacle after obstacle crossing (F_4,23_ = 10.201, p < 0.04) and lesser variability of horizontal distance from the trailing limb to the obstacle after obstacle crossing compared to control group (p < 0.01).

Regarding condition (Table 2), low obstacle showed lesser variability of step velocity compared to other obstacle height (F_2,52_ = 4,646, p < 0.001). High obstacle exhibited greater variability of leading limb toe-clearance (low obstacle – F_2,52_ = 13.381, p < 0.007 and intermediate obstacle – F_2,52_ = 13.381, p < 0.001) and lesser variability of trailing limb toe-clearance (F_2,52_ = 13.381, p < 0.001 for both obstacle height) compared to other obstacle height.

**Table 2:** Means and standard deviations (Mean ± SD) of parameters variability analyzed per condition.

		Low obstacle	Intermediate obstacle	High obstacle
Step length (%)	**GC**	4.45 ± 2.93	5.33 ± 2.43	5.89 ± 2.71
	**PD**	4.93 ± 1.80	5.19 ± 2.44	5.05 ± 1.62
Step duration (%)	**GC**	4.81 ± 1.54	5.17 ± 1.80	4.94 ± 1.15
	**PD**	4.84 ± 1.60	5.07 ± 1.77	4.91 ± 1.96
Step width (%)	**GC**	27.56 ± 9.36	32.85 ± 10.67	31.62 ± 12.56
	**PD**	30.05 ± 9.63	29.44 ± 7.27	28.83 ± 8.40
Double support (%)	**GC**	8.31 ± 2.63	8.84 ± 6.55	8.85 ± 3.09
	**PD**	6.86 ± 1.79	6.55 ± 1.16	6.82 ± 1.59
Single support(%)	**GC**	1.69 ± 0.48	1.74 ± 0.56	1.59 ± 0.62
	**PD**	1.61 ± 0.51	1.49 ± 0.34	1.47 ± 0.39
Step velocity (%)	**GC**	6.41 ± 2.19	7.76 ± 3.68^#^	7.91 ± 2.89^#^
	**PD**	6.63 ± 2.00	7.76 ± 3.40^#^	7.52 ± 2.41^#^

^#^Low obstacle is different of high and intermediate obstacle. PD: PD group; GC: Control Group.

## Discussion

The aim was to investigate the variability of spatiotemporal and kinect parameters during obstacle avoidance with different obstacle heights in people with PD and healthy neurological older people (control group). PD people showed higher values of variability than control group, mainly for horizontal distance to the obstacle and toe-clearance, corroborating with other researches^4,25^. However, the main findings discussed below are against our initial hypothesis: (i) the absence of effect in kinect parameters between groups and obstacle heights; (ii) increasing variability of step velocity during intermediate and high obstacle avoidance compared with low obstacle; (iii) the great variability of horizontal distance of trailing limb after obstacle avoidance for the control group.

Control group and people with PD seem to not variate during obstacle avoidance for kinect parameters. Both groups maintain their kinect characteristics during obstacle avoidance for all heights. A possible explanation is the robustness (hardiness) of these parameters. During aging, occurs neuromotor and muscular deteriotation^26^. This fact could be useful to understand that both group decreases muscle control and, under complexes situation (like obstacle avoidance), adopt an robustness strategy, maintaining their pattern looking for safe. Moreover, the trailing limb crossing occurs without visual information, once this member is behind participant view^8,27^ and contribute in this robustness. If we extrapolated our results and compare it with the results of Patla (1998)^28^, in which they found no difference for trailing limb during no-vision condition (regarding that they had no kinect analysis), it is possible to supposed that PD people and older people adopt this robustness strategy to keep safer. Furthermore, it is possible to link the conservation of kinect parameters before obstacle avoidance with toe-clearance (elevation of leading limb) in the three different obstacle heights. The participants maintain a safe elevation (~30 cm - the sum of obstacle height with toe clearance). In this way, it seems easier keep limb elevation height by muscle force than by the resultant of ground reaction force.

Higher and intermediate obstacles increase step velocity variability for both groups. Low obstacle avoidance showed lower values for step velocity variability than other two conditions (intermediate and high obstacle). Increase step velocity variability during obstacle avoidance could be a dangerous strategy. The physical limitation caused by higher obstacle needs a great time for preparation and attention^8^. Changing constantly the step velocity could increase fall risk, once older people did not showed muscular and motor system fully intact to react for any perturbation of the task^26^. In this form, if the planning was made on a wrong way, there will no enough time to correction and could occurring a stumbling and, consequently, a fall. Furthermore, higher obstacles generate a challenge, making more difficult to find a pattern to avoid these obstacles, increasing the variability. In other hand, low obstacles allow this pattern once it did not represent a great challenge. However, maintain the pattern could be deceptive, once both group miscalculated when they need to avoid low obstacle, let the adjustments for the last two steps before the obstacle^2^.

Horizontal foot placement to obstacle and toe-clearance are more variable in people with PD. This finding could be explained by follow arguments: (i) obstacle avoidance need higher complexity and execution of the movement^3^. People with PD present sensorial deficits^7,11^ which impair the correct environment perception. In this way, people with PD needs to perform constantly adjustments to correct errors and perform the task with successful. This situation becomes more severe in extreme situations, like low and high obstacle, increasing toe-clearance variability. The incapacity of this population to perceive the environment correctly in these situations^17^ become obstacle avoidance more challenging; (ii) obstacle avoidance relays on visual information of the relationship of the body segments with the obstacle, in the way to keep a safe distance^29^. For this, it is necessary a visuospatial attention to identify the obstacle and integrate visual and somatosensory information to elevate the foot in a safe distance to the obstacle^29^. Due to sensorial deficits of people with PD^7^, obstacle presence needs a complex interpretation, which needs more cortical areas to process the gait^11^, increasing modulation before and during obstacle avoidance; (iii) intermediate obstacle (~15 cm) is a commonly height faced off daily by participants (in Brazil, almost all curbs need to have this height, according techniques rules) and could not be so challenging as others.

Regarding our findings, the present study has some limitations. A bigger sample could be interest to reinforce our results. However, the sample size analysis indicated that the number of the actual sample is enough to present significant findings. Moreover, could be interesting to personalize the obstacle’s height according to subject’s height.

## Conclusion

People with PD present great variability than control group for horizontal distance to the obstacle and toe-clearance. The combination of task complexity and disease impairments contributing for greater variability of this group. Besides, low obstacles and high obstacle seem to exacerbate variability of horizontal distance and toe-clearance for people with PD. However, obstacle height did not interfere on kinect parameters variability during obstacle avoidance. Future studies could analyze the behavior of fallers during this task, once obstacle avoidance is the most common cause of fall among older adults.

### Ethics Committee

Older people gave informed consent by signing the informed consent form approved by the local Ethics Committee at UNESP – Campus Rio Claro/Brazil (CAAE #580.665/2013). All protocol was approved by the same Committee and was in accordance with Ethical guidelines (Brazilian Resolution #196/96 – National Health Council – National Council in Research Ethics).
