# Author Correction: Variability of crossing phase in older people with Parkinson’s disease is dependent of obstacle height

**DOI:** 10.1038/s41598-019-40318-x

**Published:** 2019-04-16

**Authors:** Lucas Simieli, Fabio Augusto Barbieri, Diego Orcioli-Silva, Ellen Lirani-Silva, Victor Spiandor Beretta, Paulo Cezar Rocha dos Santos, Lilian Teresa Bucken Gobbi

**Affiliations:** 1 São Paulo State University (Unesp) - Campus Rio Claro, Posture and Gait Studies Laboratory (LEPLO), Department of Physical Education, Rio Claro, Brazil; 2 São Paulo State University (Unesp) - Campus Bauru, Human Movement Research Laboratory (MOVI-LAB), Department of Physical Education, Bauru, Brazil

Correction to: *Scientific Reports* 10.1038/s41598-018-33312-2, published online 05 October 2018

This Article contains errors.

Figure Legends 2 and 3 were published as Figure Legends 3 and 2, the correct Figure Legends appear below:

Figure 2:

Group*obstacle variability for horizontal and vertical foot-obstacle distance before and after obstacle avoidance for leading and trailing limb. (**a**) Difference between low and high obstacle avoidance; (**b**) Difference between intermediate and high obstacle avoidance; (**c**) difference between low and intermediate obstacle. *Difference between people with PD and control group.

Figure 3:

Variability of kinects parameters for each group in each condition.

Additionally, in the Acknowledgements section:

“The author thanks to FAPESP #2014/20549-0 – Fundação de Amparo à Pesquisa do Estado de São Paulo.”

should read:

“The author thanks to FAPESP #2013/21841-3 – Fundação de Amparo à Pesquisa do Estado de São Paulo.”

